# Irradiation of *Yarrowia lipolytica* NRRL YB-567 creating novel strains with enhanced ammonia and oil production on protein and carbohydrate substrates

**DOI:** 10.1007/s00253-015-6852-2

**Published:** 2015-08-15

**Authors:** Mitch R. Lindquist, Juan Carlos López-Núñez, Marjorie A. Jones, Elby J. Cox, Rebecca J. Pinkelman, Sookie S. Bang, Bryan R. Moser, Michael A. Jackson, Loren B. Iten, Cletus P. Kurtzman, Kenneth M. Bischoff, Siqing Liu, Nasib Qureshi, Kenneth Tasaki, Joseph O. Rich, Michael A. Cotta, Badal C. Saha, Stephen R. Hughes

**Affiliations:** 1 United States Department of Agriculture (USDA), Agricultural Research Service (ARS), National Center for Agricultural Utilization Research (NCAUR), Renewable Product Technology Research Unit, 1815 North University Street, Peoria, IL 61604 USA; 2 National Coffee Research Centre – Cenicafe, National Federation of Coffee Growers of Colombia - FNC, Cenicafé Planalto Km 4 vía Antigua Chinchiná, Manizales, Caldas Colombia; 3 4160 Department of Chemistry, Illinois State University, 214 Julian Hall, Normal, IL 61790-4160 USA; 4 South Dakota School of Mines & Technology, Chemical and Biological Engineering, 501 East Saint Joseph Street, Rapid City, SD 57701-3995 USA; 5 USDA, ARS, NCAUR, Bio-oils Research Unit, 1815 North University Street, Peoria, IL 61604 USA; 6 USDA, ARS, NCAUR, Bioenergy Research Unit, 1815 North University Street, Peoria, IL 61604 USA; 7 USDA, ARS, NCAUR, Bacterial Foodborne Pathogens and Mycology Research Unit, 1815 North University Street, Peoria, IL 61604 USA; 8 Mitsubishi Chemical, USMC Research & Innovation, 410 Palos Verdes Blvd, Redondo Beach, CA 90277 USA

**Keywords:** *Yarrowia lipolytica* UV-C mutagenesis, *Yarrowia* protein utilization, Acylglycerols from oleaginous yeast, *Yarrowia* ammonia production, *Yarrowia* carbohydrate substrate utilization

## Abstract

Increased interest in sustainable production of renewable diesel and other valuable bioproducts is redoubling efforts to improve economic feasibility of microbial-based oil production. *Yarrowia lipolytica* is capable of employing a wide variety of substrates to produce oil and valuable co-products. We irradiated *Y. lipolytica* NRRL YB-567 with UV-C to enhance ammonia (for fertilizer) and lipid (for biodiesel) production on low-cost protein and carbohydrate substrates. The resulting strains were screened for ammonia and oil production using color intensity of indicators on plate assays. Seven mutant strains were selected (based on ammonia assay) and further evaluated for growth rate, ammonia and oil production, soluble protein content, and morphology when grown on liver infusion medium (without sugars), and for growth on various substrates. Strains were identified among these mutants that had a faster doubling time, produced higher maximum ammonia levels (enzyme assay) and more oil (Sudan Black assay), and had higher maximum soluble protein levels (Bradford assay) than wild type. When grown on plates with substrates of interest, all mutant strains showed similar results aerobically to wild-type strain. The mutant strain with the highest oil production and the fastest doubling time was evaluated on coffee waste medium. On this medium, the strain produced 0.12 g/L ammonia and 0.20 g/L 2-phenylethanol, a valuable fragrance/flavoring, in addition to acylglycerols (oil) containing predominantly C16 and C18 residues. These mutant strains will be investigated further for potential application in commercial biodiesel production.

## Introduction

One of the major challenges facing commercial production of biofuels and bioproducts is cost-effective utilization, detoxification, and processing of biomass and other inexpensive carbon sources such as coffee and fruit processing wastes and other agricultural and food waste. The efficient conversion of low-cost substrates to advanced biofuels requires development of improved microbial catalysts (Hughes and Riedmuller 2014; Koutinas et al. 2014; Peralta-Yahya et al. 2012). Economic feasibility of biosynthetic fuel and chemical production depends on optimization of these biocatalysts to achieve high yields of the desired products. *Saccharomyces cerevisiae* is currently the most employed microbial catalyst in the biotechnology industry, but this yeast is limited in its range of substrates for producing fuel ethanol, and although genetic engineering has improved its utilization of the constituent pentose sugars of lignocellulosic materials, development of a recombinant *S. cerevisiae* strain capable of efficient pentose utilization remains a challenge (Casey et al. 2013; Garcia Sanchez et al. 2010; Hughes et al. 2009a, b; Kim et al. 2013a, b; Matsushika et al. 2014; Nielsen et al. 2013; Oreb et al. 2012; Zhou et al. 2012). Other microbial catalysts are being investigated for the production of biofuels and value-added bioproducts. One candidate is the oleaginous yeast species *Yarrowia lipolytica*, which has the potential for producing advanced biofuels and chemicals from agricultural and food waste (Abghari and Chen 2014; Blazeck et al. 2014; Groenewald et al. 2014; Harzevili 2014; Hughes et al. 2014; Tsigie et al. 2011; Xu et al. 2013).

Interest in *Y. lipolytica* initially arose from its uncommon physiological characteristics. Strains of this species were more often isolated from lipid- or protein-containing substrates like cheese or sausage than from sugar-containing substrates. It secretes several metabolites in large amounts, such as organic acids and extracellular proteins, and the tools are available for expression and secretion of heterologous proteins (Barth and Gaillardin 1996, 1997; Nicaud et al. 2002). *Y. lipolytica* is widely utilized in industrial applications such as extracellular enzyme production (lipases, alkaline or acid proteases, phosphatases) (Harzevili 2014), organic acid biosynthesis, including citric (Papanikolaou et al. 2009; Sauer et al. 2008) and alpha-ketoglutaric (Morgunov et al. 2013; Otto et al. 2012), cheese ripening (Mansour et al. 2008), and single cell oil (SCO) production (Beopoulos et al. 2009; Huang et al. 2013). It is similar to *Escherichia coli* and *S. cerevisiae* in ease of manipulation and growth capacity. It is also able to perform post-translational processing of complex proteins, has a mainly co-translational secretion pathway, high secretion capacity and product yield, and low hyperglycosylation of products. Furthermore, production scale-up is relatively simple, giving it advantages as a protein expression system (Blazeck et al. 2011; Gasmi et al. 2011; Madzak et al. 2004; Madzak and Beckerich 2013). In addition, the whole genome of *Y. lipolytica* has been sequenced (Dujon et al. 2004).

*Y. lipolytica* is being studied for removal of sugars and proteins from microbial fermentation waste to make oil and protein or to produce amino acids for animal feed or food additives or the flavoring and fragrance 2-phenylethanol (Celińska et al. 2013), or for the biosynthesis of new products, such as erythritol and mannitol, whose synthesis from glycerol by *Y. lipolytica* would have advantages over their production from common sugars (Rywińska et al. 2013; Tomaszewska et al. 2012). Among the compounds produced by *Y. lipolytica* are omega-3 fatty acids for use as health supplements and in the pharmaceutical, aquaculture, animal feed, pet food, and personal care markets (Berge et al. 2013; Xue et al. 2013), alpha-ketoglutaric, pyruvic, isocitric, citric, and succinic acids using n-alkanes, glucose, and glycerol as carbon sources (Finogenova et al. 2005; Otto et al. 2013; Rywińska et al. 2013; Yuzbashev et al. 2010), and citric acid and single cell oil from glycerol waste (Papanikolaou and Aggelis 2009). Blazeck and coworkers (2014) undertook extensive genotypic and phenotypic optimization of the native metabolism of *Y. lipolytica* to create a strain that enhanced lipid accumulation in *Y. lipolytica* by >60-fold compared to the starting strain. Furthermore, they demonstrated these lipids can be readily converted into fatty acid methyl esters suitable for biodiesel, supporting the potential of *Y. lipolytica* as a platform for sustainable production of biodiesel and other important oleochemicals (Blazeck et al. 2014).

The development of *Y. lipolytica* as a platform will require obtaining more naturally versatile strains able to utilize a wide variety of substrates, enhancing yield and productivity on inexpensive feedstocks, improving fermentation and separation processes in favor of the modified strain, and genetically engineering the optimized strain for production of biofuels and bioproducts (Abghari and Chen 2014; Blazeck et al. 2014; Huang et al. 2013). The selection of the optimal *Y. lipolytica* strain through screening is one of the most important needs in industrial applications because *Y. lipolytica* strains differ in terms of biomass and bioproduct production rates under similar conditions (Bordes et al. 2007; Etschmann et al. 2003; Satpute et al. 2010).

The yeast strain *Y. lipolytica* NRRL YB 567 was selected for the present study because of its ability to utilize protein to produce ammonia (Gardini et al. 2001; Ismail et al. 2000; Kurtzman 2011; Mansour et al. 2008). Protein is a major constituent of fermentation residues in a biorefinery and is present at a concentration of about 11 % in coffee processing waste (Elías 1979). Bio-based ammonia is a valuable sustainable fertilizer (Steinberg 2006). In order to improve the amount of ammonia and oil it produces, *Y. lipolytica* NRRL YB-567 was irradiated with UV-C as described previously for *Kluyveromyces marxianus* NRRL Y-1109 (Hughes et al. 2013) and subsequently screened for increased ammonia and triglyceride production. The focus was to obtain *Y. lipolytica* host strains that produce high levels of ammonia and oil for improved renewable gasoline and biodiesel production and that can also be engineered to express genes concomitantly for value-added bioproducts (Beopoulos et al. 2009; Madzak and Beckerich 2013). The ammonia and oil produced by the novel strains have potential industrial applications such as bio-based fertilizer or as renewable drop-in replacements for gasoline or as third generation biodiesel fuel.

## Materials and methods

### Culture conditions

*Yarrowia lipolytica* NRRL strains YB-271, YB-387, YB-423-12, YB-437, YB-567, and Y-1095 were obtained from the USDA ARS Culture Collection, spread onto YPD plates [10 g yeast extract, 20 g dextrose, 20 g peptone, and 20 g Bacto®Agar (Becton, Dickinson and Company, Franklin Lakes, NJ) per liter deionized water] and incubated at 30 °C for 24 h. The colonies were then transferred to separate 15-mLpolycarbonate conical tubes containing 5 mL of liver (no glucose) (LNG) liquid medium [35 g of Difco liver infusion broth (Becton, Dickinson and Company, Franklin Lakes, NJ) per 1 liter deionized water] and incubated at 30 °C for 24 h. The liquid cultures were adjusted to an optical density (OD_660_) of 0.5 (Beckman DU-640). Ten microliters of each strain were spotted onto an LNG plate [35 g of Difco liver infusion broth and 20 g of Bacto Agar (Becton, Dickinson and Company, Franklin Lakes, NJ) per 1 liter deionized water; autoclaved for 20 min at 250 °F]. The plates were incubated at 30 °C for 24 h. Strain *Y. lipolytica* NRRL YB-567 was selected for irradiation because it exhibited the greatest amount of growth on LNG medium.

Simulated coffee waste medium was prepared using 60 % pulp and 40 % mucilage (dry, milled; 12.5 % total solids) in distilled water; autoclaved for 10 min at 120 °C and 15 psi, centrifuged at 3000 rpm for 10 min, and the supernatant liquid fractions autoclaved again for 10 min at 120 °C and 15 psi; and 20 g/L Bacto Agar were added for agar plates. For experiments with borra, 20 % (*w*/*w*) powdered borra (spent coffee grounds) was added. For *K. marxianus*-treated coffee waste, the mixture was treated with *K. marxianus* mutant strain NRRL Y-50798 (Hughes et al. 2013) in a 30-L fermentation at 30 °C for 3 days at 100 rpm. The major components of coffee waste pulp and mucilage are fructose 28 %, cellulose 20 %, glucose 18 %, lignin 12 %, and protein 11 %; Elías 1979).

### Irradiation of Y. lipolytica NRRL YB-567

**Fig. 1:**
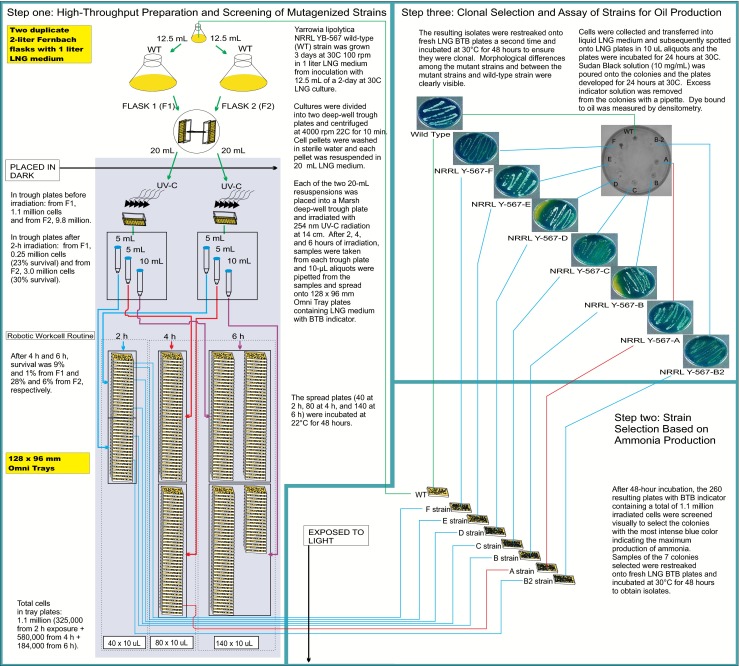
Steps in the procedure for mutagenesis of *Yarrowia lipolytica* NRRL YB-567 and screening to select mutant strains *A*, *B*, *B2*, *C*, *D*, *E*, and *F*

The irradiation method used (Fig. 1, step 1) was similar to that previously used by Hughes and coworkers (2013) for producing mutagenized *K. marxianus* strains, except that a wavelength of 254 nm instead of 234 nm was used, and the radiation exposure time was adjusted to achieve the desired target of at least 70 % of cells killed. Duplicate 2-L Fernbach flasks were prepared by adding 1 L of LNG medium to each flask and inoculating with 12.5 mL of a culture equivalent to OD_660_ = 20 of wild-type *Y. lipolytica* NRRL YB-567 grown on 30 mL of LNG medium in a 125-mL flask at 30 °C for 2 days. The Fernbach flasks were incubated at 30 °C for 3 days at 100 rpm. The cultures were divided into two deep-well trough plates with baffled bottoms (E&K Scientific Products, Inc., Santa Clara, CA; http://www.eandkscientific.com/96-Individual-Deep-Well-Reservoir-Pyramid-Bottom-300ml.html) and centrifuged at 4000 rpm in a Beckman Avanti J-20 XP centrifuge with swinging bucket rotor JS-4.3 (Beckman Coulter, Inc., Indianapolis, IN) at 22 °C for 10 min. The supernatant was decanted, the cell pellets were washed, and the pellets from each Fernbach flask were resuspended in 20 mL of LNG medium in two deep-well trough plates. Before irradiation, a 10-μL sample of the resuspension derived from each of the Fernbach flasks was taken, diluted to 10^-3^, plated, and an initial cell count obtained. The cells in each trough plate were irradiated with 254 nm UV-C radiation [UVP, LLC Light Table (inverted; Upland, CA)] at a distance of 14 cm above the trough plates. After 2 h of irradiation, 5-mL samples were taken from each trough plate and 10-μL aliquots spread onto twenty 128 × 96 mm Omni Tray plates (Thermo Fisher Scientific, Waltham, MA) for a total of 40 tray plates from both trough plates. After 4 h, 5-mL samples were again taken from each trough plate and 10-μL aliquots spread onto 40 tray plates for a total of 80 tray plates from both trough plates. After 6 h, the remaining 10 mL was taken from each trough plate and 10-μL aliquots from the trough plate from Fernbach flask 1 were spread onto 80 tray plates and 10-μL aliquots from the trough plate from Fernbach flask 2 were spread onto 60 tray plates for a total of 140 tray plates from both trough plates. A total of 1.1 × 10^6^ cells were screened, 3.3 × 10^5^ cells on the 40 tray plates from the 2-h irradiation, 5.8 × 10^5^ cells on the 80 tray plates from the 4-h irradiation, and 1.8 × 10^5^ cells on the 140 tray plates from the 6-h irradiation.

An automated protocol on a robotic workcell (Hughes et al. 2005) was used to spread the 10-μL aliquots from these samples onto the 128 × 96 mm Omni Tray plates. The plates contained LNG medium with the pH indicator bromothymol blue (BTB) [35 g of Difco liver infusion broth (Becton, Dickinson and Company, Franklin Lakes, NJ), 0.1 g BTB (Sigma Aldrich, St. Louis, MO), and 20 g Bacto Agar (Becton, Dickinson and Company, Franklin Lakes, NJ) per liter]. The tray plates from several passive and active stackers were moved to the liquid handler in a scheduled fashion where they were spotted with medium and then with irradiated culture from the deep-well trough plates on the deck. The tray plates (40 at 2 h, 80 at 4 h, and 140 at 6 h) were incubated for 48 h at 22 °C. The kill curve was determined by taking a 10-μL sample from the trough plates after irradiation for 2, 4, and 6 h, diluting as necessary, plating, and counting the surviving cells on the plate.

### Indicator for ammonia production

Bromothymol blue (also known as bromthymol blue; BTB) is a commonly used pH indicator and was selected for ammonia screening because of its easily observed color change from yellow to blue at pH 7.6. The plate medium was prepared using 35 g of Difco liver infusion broth, 20 g of Bacto Agar, and 0.1 g of BTB indicator per liter deionized water. The pH of the resulting medium was adjusted to 4.5 using 3 N HCl. The growth of *Y. lipolytica* NRRL YB-567 on the plate corresponded to a color change of yellow to blue indicating an increase in pH above 7.6 (more alkaline).

### Screen for ammonia production

After incubation of the tray plates for 48 h, they were screened visually to select the colonies that produced the most intense blue color with the indicator. Seven mutant strains, six from 2-h exposure, one from 4-h exposure, and none from 6-h exposure, were selected, and samples of these seven colonies were re-streaked onto seven fresh LNG BTB plates and incubated at 30 °C for 48 h to obtain isolates (Fig. 1, step 2). The resulting isolates were re-streaked a second time onto fresh LNG BTB plates and incubated at 30 °C for 48 h to ensure they were clonal (Fig. 1, Step 3).

### Densitometry for determination of oil

A 10-μL aliquot of each of the mutant strains and the wild-type strain was spotted on a plate containing LNG medium and incubated for 24 h at 30 °C. Five mL of Sudan Black B indicator in LNG medium (10 mg/mL; vortexed) were layered over the colonies on the plate and incubated at 30 °C for 24 h. Sudan Black B indicator solution that was not bound to the oil (lipids) was siphoned off the plate with a pipette (Fig. 1, step 3). Densitometry measurements were performed in duplicate to determine the amount of indicator bound to each strain using an AlphaImager® 3400 (Alpha Innotech Corporation, San Leandro, CA), and the values (as percent of wild-type) were calculated.

### Scanning electron micrographs

Cells from *Y. lipolytica* wild-type and mutant strains grown in 5 mL liquid LNG medium inoculated with 10 μL of a 0.1 OD culture in LNG and incubated for 24 h at 30 °C and 100 rpm were harvested, suspended in saline (0.85 % NaCl), and centrifuged to remove residual medium. Following a modified procedure of Bang and Pazirandeh (1999), the cell pellet was suspended and fixed in 2.5 % glutaraldehyde prepared in 100 mM cacodylate buffer, pH 7.2, for 1 h on ice. To remove remaining glutaraldehyde, the cells were rinsed with cacodylate buffer twice and with distilled water once, allowing several minutes for each step. The cells were dehydrated, respectively, in solutions containing 50, 60, 70, 80, 90, and 100 % ethanol successively for 15 min for each treatment. Cells were mounted on an aluminum stub and placed in a desiccator to dry overnight or until needed. The samples were examined using the Zeiss Supra40 Variable-Pressure Field-Emission Scanning Electron Microscope (Engineering and Mining Experiment Station, Rapid City, SD) at an accelerating voltage of 1 kV, and the subsequent images were analyzed to evaluate cell morphology.

### Light microscopy

*Y. lipolytica* wild-type and mutant strains were grown at 30 °C for 5 days on simulated coffee waste medium. Colonies were examined under the Leica Z16 APO zoom microscope (Engineering and Mining Experiment Station, Rapid City, SD), and the subsequent images were analyzed to evaluate colony morphology.

### Variable nucleotide tandem repeat (VNTR) PCR

Variable nucleotide tandem repeat (VNTR) PCR analysis was performed to detect differences in genomic DNA sequences between wild-type and mutant strains using as both the forward and reverse PCR primers the 28-base pair (bp) repeating unit of the highly polymorphic VNTR sequences present in the 3′ flanking region of the *HRAS* gene: 5′AGG GGA CGC CAC ACT CGC CCT TCT CTC C3′ (Nakamura et al. 1998). Each strain was cultured in 100 mL LNG liquid medium, incubated at 30 °C for 2 days, and the cells were pelleted by centrifugation at 4,000×*g* for 10 min. The pellet was frozen using liquid nitrogen and ground into a fine powder with a mortar and pestle. The powder was transferred into a 15-mL conical tube with a spatula. Extraction of the DNA was performed using the Qiagen DNeasy Plant Genomic kit (Qiagen, Inc., Valencia, CA) according to the manufacturer’s directions. The concentration of DNA obtained was 1.0 μg/μL determined spectrophotometrically (Beckman DU 800; Beckman; Indianapolis, IN) using absorbances at 260 and 280 nm.

The PCR mixture contained 4 μL DNA, 34 μL H_2_O, 10 μL 5X Phusion HF Buffer, 1 μL 10 mM dNTPs, 0.5 μL (1 mg/mL) VNTR oligonucleotide, and 0.5 μL Phusion Enzyme (Finnzymes Phusion High-Fidelity PCR kit; New England Biolabs, Ipswich, MA). The PCR reaction was prepared in a Bio-Rad hard-shell 96-well PCR plate (Bio-Rad Laboratories, Hercules, CA) on ice and was carried out in a PTC-225 Tetrad Thermal Cycler (Bio-Rad Laboratories, Hercules, CA) using the following conditions: hold at 96 °C for 5 min, cycle at 96 °C for 1 min, 65 °C for 1 min, 72 °C for 1 min, repeated for 30 times, followed by 72 °C for 7 min and a 4 °C hold. The procedure amplified the genomic sequence between two VNTR sequences to determine alterations in the minisatellite regions in the genome caused by UV-C mutagenesis. The amplified DNA was analyzed by gel electrophoresis on 1 % (*w*/*v*) agarose gels stained with ethidium bromide using lambda DNA-*Hin*d III/phiX-174 RF DNA-*Hae* III marker (72 bp to 23 kb; Thermo Fisher Scientific, Waltham, MA). Electrophoresis was performed at 100 volts for 1.5 h on a Bio-Rad Power Pac 3000, and a high-resolution digital image file was generated with an AlphaImager 3400 using a trans-UV light (Alpha Innotech Corporation, San Leandro, CA).

### Coomassie SDS PAGE analysis

Liquid cultures were prepared in YPD medium in 5-mL conical tubes and incubated for 3 days at 30 °C and 100 rpm shaking in a New Brunswick Innova 4240 shaker (Eppendorf, Inc., Enfield, CT). One mililiter of culture was placed in a 1.5-mL microcentrifuge tube, centrifuged for 2 min at 13,000 rpm, and the supernatant transferred to another tube. Twenty mililiter of 2× tris-glycine loading buffer (pH 8.3; Invitrogen) plus 2 mL beta-mercaptoethanol (Bio-Rad Laboratories, Hercules, CA) were prepared, and 40 μL added to the cell pellet. The sample was vortexed thoroughly and heated at 95 °C for 10 min. Ten microliters of sample were loaded onto a 4 to 20 % tris-glycine SDS-PAGE gel (Invitrogen, Life Technologies, Grand Island, NY) in an XCell Novex box and run in 1× tris-glycine running buffer (Invitrogen) at 125 V for 100 min on the Bio-Rad PowerPac Basic (Bio-Rad Laboratories, Hercules, CA). Ten μL SeeBlue® Plus2 marker (Invitrogen, Life Technologies, Grand Island, NY) were loaded in the first lane. After electrophoresis, the gel was stained for 1 h with Coomassie Brilliant Blue R-250 staining solution (Bio-Rad Laboratories, Hercules, CA) and destained for 20 h with a solution of 15 % (*v*/*v*) methanol and 10 % (*v*/*v*) acetic acid. The image of the gel was obtained using the AlphaImager® System 3400 (Alpha Innotech Corporation, San Leandro, CA).

### Ammonia quantitation assay

A Megazyme Ammonia Assay kit (Megazyme International, Ireland) was used to determine the amount of ammonia produced by each strain. The strains were cultured in 60 mL of LNG liquid medium (3.5 % Difco Liver infusion broth in water) in 125-mL flasks incubated at 30 °C and 100 rpm. At 24, 48, 72, 96, 120, and 144 h, 5 mL were removed from each culture and added to 15-mL conical tubes (Sigma-Aldrich, St. Louis, MO). In order to decolorize the samples, 0.1 g poly(vinylpolypyrrolidone) was added to each tube. The samples were vortexed for 5 min and filtered through Whatman No.1 filter paper. Protein was removed from the recovered samples by adding an equal volume of 1 M perchloric acid to each and centrifuging at 1500×*g* for 10 min. The supernatant was neutralized using 10 M KOH and centrifuged again at 1500×*g* for 4 min. A quantity of 0.1 mL of each supernatant was added to a 3-mL polystyrene cuvette that contained 2.0 mL water, 0.3 mL buffer solution as supplied (pH 8.0, with 2-oxoglutarate and sodium azide (0.02 %) as preservative), and 0.2 mL NADPH solution prepared as directed in the kit. The blank contained 2.1 mL water, 0.3 mL buffer solution, and 0.2 mL NADPH solution. The cuvettes were sealed with Parafilm and mixed by gentle inversion. The absorbances of the sample, blank, and ammonia standard at 340 nm were determined using a Beckman DU 800 (Beckman; Indianapolis, IN). After adding 0.02 mL glutamate dehydrogenase suspension (as supplied, swirled), the solutions were mixed and allowed to stand 5 min before taking a second reading. The concentration (g/L) of ammonia (*c*) in the sample is calculated using the equation: *c* = [(*V* × *MW*) / (*ε* × *d* × *v*)] [(*A*_*1*_ − *A*_*2*_)_*s*_ − (*A*_*1*_ − *A*_*2*_)_*b*_] where *V* is final volume in mL, *MW* is the molecular weight of ammonia in g/mol, *ε* is extinction coefficient of NADPH at 340 nm (6300 L mol^−1^ cm^−1^), *d* is light path in cm, *v* is the added supernatant volume in mL, and *A*_*1*_ and *A*_*2*_ are the absorbance readings of the sample (*s*) and blank (*b*) before addition of glutamate dehydrogenase and at the end of the reaction, respectively.

### Bradford protein assay

Protein concentrations were determined with a Bio-Rad Protein Assay kit (Bio-Rad Laboratories, Hercules, CA) using the standard protocol based on the Bradford dye-binding method (Bradford 1976). A 30-μL sample was added to 1.5 mL of Coomassie Reagent (Coomassie Brilliant Blue G-250 dye) prepared as directed and vortexed. Samples were incubated at room temperature for 5 min and the absorbance was measured at 595 nm. Concentrations of the samples were obtained from a standard curve generated by using three dilutions of a BSA standard as described in the protocol.

### Fermentation on coffee waste medium

The appropriate coffee waste medium was added to a 125-mL flask and inoculated with 20 mL of *Y. lipolytica* mutant strain F at 0.1 OD_660_. The medium was incubated for 7 days and tested each day for ammonia and protein production.

### Determination of fatty acid composition of oil

Fatty acid composition was determined by derivatization of oil with methanolic KOH to generate fatty acid methyl esters (FAMEs) as described in Ichihara et al. (1996) and analyzed using a PerkinElmer (Waltham, MA) Clarus 580 GC equipped with FID, built-in autosampler and HP88 column (30 m × 0.25 mm i.d., 0.20 μm film thickness). The carrier gas was H_2_ with a flow rate of 15.0 mL/min. The temperature program was as follows: hold at 100 °C for 5 min, ramp from 100 °C to 220 °C at 10 °C/min and hold at 220 °C for 15 min. Injection volume was 1.0 μL with a split ratio of 10.0:1.0. The concentration of sample in hexane (1 mL) was approximately 20 mg/mL. The injector and detector temperatures were 240 and 280 °C, respectively. FAME peaks were identified (triplicates; means reported) by comparison to reference standards (Nu Chek Prep, Elysian, MN).

### Determination of triglyceride concentrations

The concentrations of monoacylglycerols (MAGs), diacylglycerols (DAGs), and triacylglycerols (TAGs) were determined using an Agilent (Santa Clara, CA) 7890 GC equipped with FID, Agilent model 7683B auto sampler, and Agilent J & W DB-5HT column (15 m × 0.320 mm i.d., 0.10 μm film thickness) with H_2_ as carrier gas. The temperature program as well as peak identification and quantification were performed as described in ASTM D6584 (ASTM 2013).

### Isolation and quantitation of 2-phenylethanol and phenol

Cell suspensions were withdrawn at daily intervals and stored at −80 °C prior to analysis. The suspensions were centrifuged to remove cells (13,000 rpm, 10 min). Two milliliters of the supernatant were loaded onto a 1 g C18 sample preparation cartridge (Alltech Associates, Inc., Deerfield, IL). This was then washed with 2.0 mL water, and the 2-phenylethanol (2PE) and phenol were eluted with 2.0 mL methanol. Analysis was performed on a Shimadzu QP2010 SE GC/mass spectrometer/FID (Shimadzu Scientific Instruments, Columbia, MD). Separations were accomplished using a Supelco Petrocol DH 50.2 (50 m × 0.2 mm id; 0.5 μm film thickness) column for separations (Sigma-Aldrich, St. Louis, MO). The oven program was as follows: initial temperature 120 °C for 2 min, ramp at 20 °C/min to 180 °C, and ramp at 25 °C/min to 300 °C with a final hold time of 6.2 min. Methyl nonanoate was used as internal standard. The mass spectrometer was operated in the EI mode at 70 eV.

## Results

### Production of Yarrowia lipolytica NRRL YB-567 mutant strains

When *Y. lipolytica* NRRL YB-567 cells from the cultures in the trough plates from the two Fernbach flasks were irradiated with 254 nm UV-C as depicted in Fig. 1, step 1, the target of >70 % cell mortality was achieved in 2 h, with the number of cells decreasing from 1.1 × 10^6^ cells in the trough plate from flask 1 and 9.8 × 10^6^ cells in the trough plate from flask 2 initially to 2.5 × 10^5^ cells and 3.0 × 10^6^ cells at 2 h, respectively, giving cell mortalities of 77 and 70 %. At 4 h, cell mortalities were 91 and 72 % for trough plates from flask 1 and flask 2, respectively. At 6 h, cell mortalities were 99 and 94 % for trough plates from flask 1 and flask 2, respectively. Samples of the surviving cells were taken from each trough plate after 2, 4, and 6 h of irradiation, spread on tray plates containing LNG BTB indicator medium, and incubated at 22 °C for 48 h. Based on percent survival, a total of 1.1 × 10^6^ cells were present on the 260 tray plates, 3.3 × 10^5^ cells on the 40 tray plates from the 2-h irradiation, 5.8 × 10^5^ cells on the 80 tray plates from the 4-h irradiation, and 1.8 × 10^5^ cells on the 140 tray plates from the 6-h irradiation. The tray plates were screened visually to select colonies with the most intense blue color indicating high levels of ammonia production. The results are depicted in Fig. 1, step 2. Seven colonies, six from 2-h exposure, one from 4 h, and none from 6 h, were selected. Samples of these colonies were re-streaked onto seven fresh LNG BTB indicator plates and incubated at 30 °C for 48 h to obtain isolates. The resulting isolates were re-streaked a second time onto fresh LNG BTB plates and incubated at 30 °C for 48 h to ensure they were clonal as pictured in the photographs in Fig. 1, step 3. The resulting mutant strains selected for ammonia production were designated A (from 4-h exposure), B, B2, C, D, E, and F (from 2-h exposure). These strains were also evaluated for oil (lipid) production using Sudan Black B indicator as pictured in the photograph in Fig. 1, step 3. Mutant strain F produced the darkest black area with this indicator.

### Morphology

**Fig. 2:**
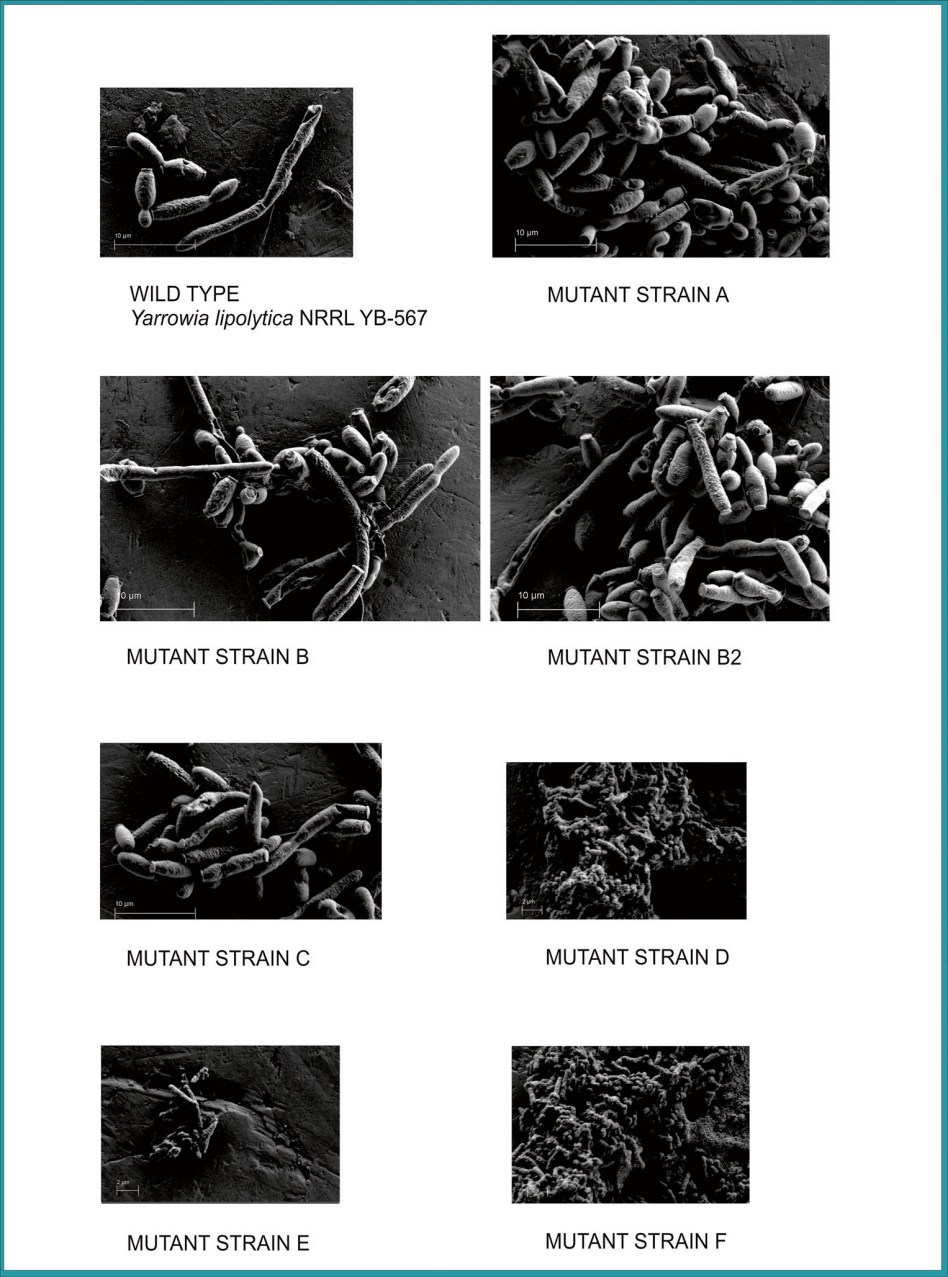
Scanning electron micrographs of *Yarrowia lipolytica* mutant strains *A*, *B*, *B2*, *C*, *D*, *E*, and *F* compared to wild-type strain (*Yarrowia lipolytica* NRRL YB-567) grown on LNG medium. Scale is indicated by a *bar* in the lower left corner of each micrograph

**Fig. 3:**
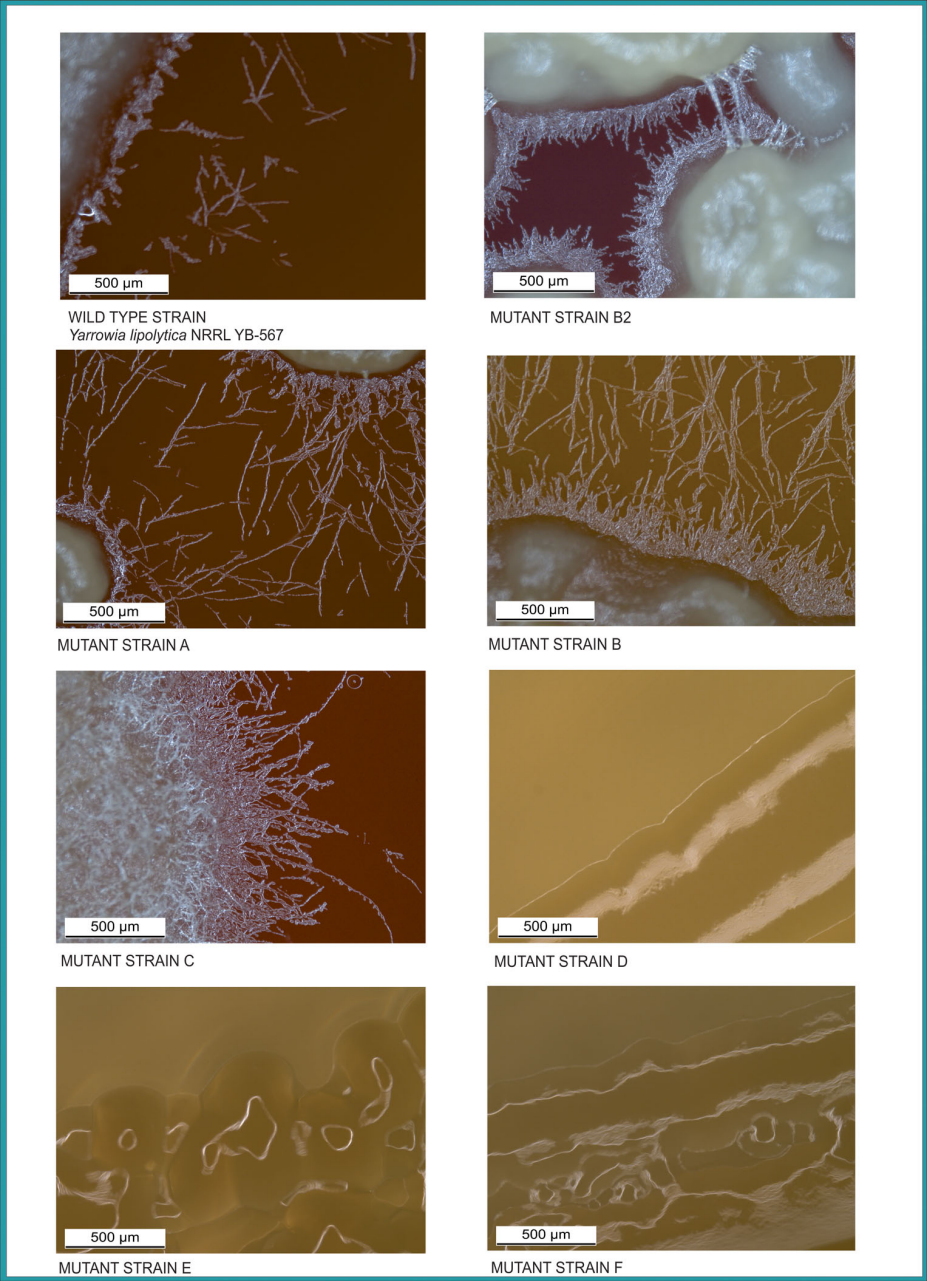
Light microscope photographs of *Yarrowia lipolytica* mutant strains A, B, B2, C, D, E, and F compared to wild-type strain (*Yarrowia lipolytica* NRRL YB-567) grown on simulated coffee waste medium. Scale is indicated by *bar* in lower left corner of each photograph

Scanning electron micrographs and light microscopy photographs (Figs. 2 and 3, respectively) show the morphological differences in the mutant strains when compared to the wild-type strain and to one another. The scanning electron micrographs (Fig. 2) show cells from *Y. lipolytica* wild-type and mutant strains in liquid LNG medium inoculated with a culture in LNG medium and incubated for 24 h at 30 °C and 100 rpm. The most notable morphological differences were the much smaller cell size (yeast-like forms about 1–2 μm in length) of mutant strains D, E, and F compared to the other mutant strains, A, B, B2, and C, and to the wild-type strain (yeast forms about 5 μm in length) and the bumpier cell surface of mutant strain F compared to the other strains. The cells of the wild-type strain exhibited both yeast (mostly ellipsoidal) and pseudohyphal forms with the yeast forms similar in size and shape. The surfaces of the yeast forms were smoother than the pseudohyphal projections, and the cells had budding scars at the polar ends. Cells of mutant strain A varied widely in size and shape; mostly, yeast and pseudohyphal forms with budding scars at the ends were present, although a few somewhat distorted shapes were also seen. The surfaces of most cells were very slightly bumpy, although a few of the yeast forms had relatively smooth surfaces. The cells of mutant strain B consisted of numerous very elongated forms and a few other distorted shapes. Both yeast and pseudohyphal forms were present, and some smooth and some bumpy surfaces were observed for all forms. The cells of mutant strain B2 had the largest variety of shapes and sizes, including elongated, distorted, yeast, and pseudohyphal forms, mostly smooth, but also some with slightly bumpy and a few with very bumpy surfaces. Cells of mutant strain C were mainly yeast and pseudohyphal forms with a few distorted elongated forms; almost all have bumpy surfaces. Cells of mutant strains D and F showed extensive branching and were intertwined forming a layer-like assembly; the surfaces of all cells were very bumpy. It was difficult to distinguish individual yeast and pseudohyphal forms, although both seemed to be present. The cells of mutant strain E were all misshapen yeast and pseudohyphal forms with extremely irregular surfaces.

Light microscopy images of *Y. lipolytica* wild-type and mutant strains grown on plates containing simulated coffee waste medium for 5 days at 30 °C are shown in Fig. 3. The strains differed widely in the amount and length of the pseudohypae observed. Mutant strain C displayed substantially more, mostly longer, pseudohyphae than the wild-type or other mutant strains. Mutant strains A and B had numerous pseudohyphae, both had more than wild type. Among the strains with observable pseudohyphae, those of mutant strain B2 were much shorter than A, B, C, or wild type. Mutant strains D, E, and F appeared to have no distinguishable pseudohyphae.

### VNTR analysis of genomic DNA

**Fig. 4:**
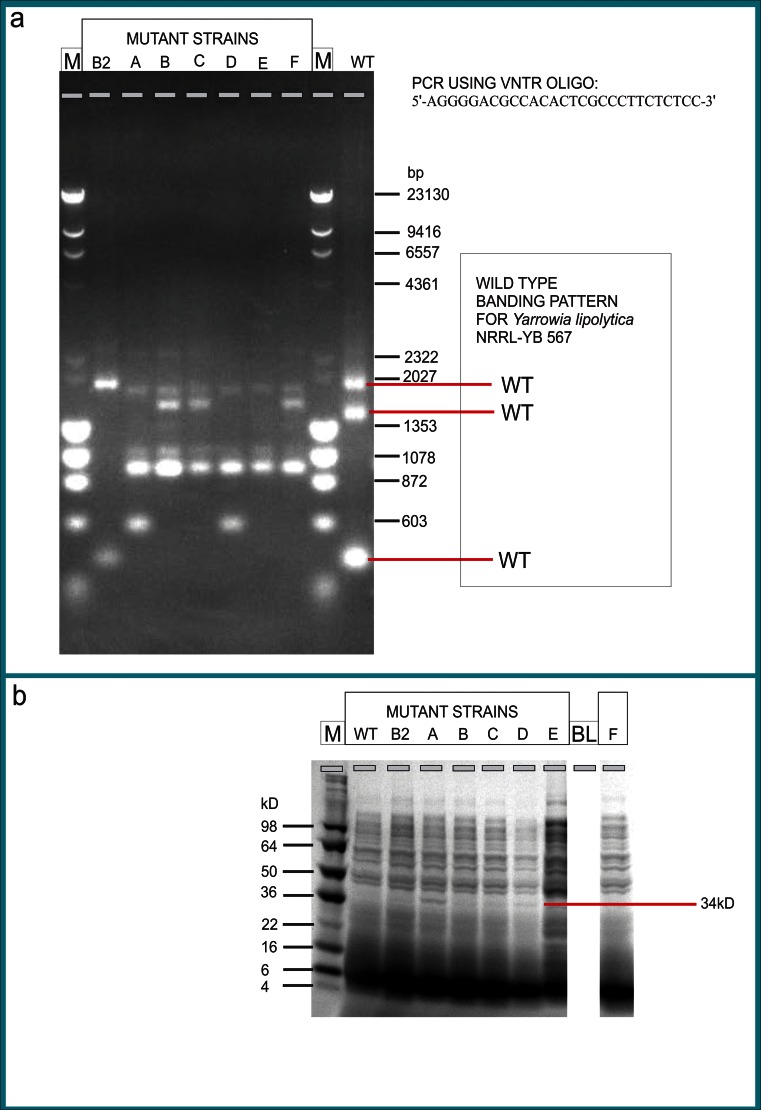
Variable nucleotide tandem repeat (VNTR) and Coomassie SDS PAGE analyses of *Yarrowia lipolytica* wild-type and mutant strains *A*, *B*, *B2*, *C*, *D*, *E*, and *F*. **a** Genomic DNA extracted from strains grown in LNG medium was amplified by PCR using the VNTR oligonucleotide indicated at upper right and the amplified DNA loaded onto 1 % (*w*/*v*) agarose gels stained with ethidium bromide. Marker in lanes labeled M is lambda DNA-*Hin*d III/phiX-174 RF DNA-*Hae* III (72 bp to 23 kb); *Y. lipolytica* mutant strains are in lanes labeled *B2*, *A*, *B*, *C*, *D*, *E*, and *F*; wild-type strain is in lane *WT*. **b** Cell protein profile of samples prepared from cultures grown in YPD medium. Samples were loaded onto a 4 to 20 % tris-glycine SDS-PAGE gel and gel was stained with Coomassie Brilliant Blue R-250. Lane *M* is SeeBlue® Plus2 marker; *Y. lipolytica* mutant strains are in lanes labeled *B2*, *A*, *B*, *C*, *D*, *E*, and *F*; wild-type strain is in lane *WT*

The PCR products amplified from the genomic DNA of *Y. lipolytica* NRRL YB-567 mutant strains using a variable nucleotide tandem repeat (VNTR) primer produced different bands (varying multiples of the 28 bp repeating unit) from those of the wild-type strain when analyzed on an agarose gel (Fig. 4a). The wild-type strain had strong bands at approximately 2020, 1450, and 500 bp. The mutant strains, with the exception of mutant strain B2, all had a strong band at approximately 950 bp and weaker bands at about 1080 and 2000 bp that were not present in the wild-type strain, and they had no bands corresponding to the three bands in wild type. Mutant strain B2 had only two strong bands (2020 and 500 bp) both of which appeared in the profile of wild type; however, the band at 500 bp was weaker and the third band seen in the wild-type strain (at 1450 bp) was not present. In addition to having the three bands (2000, 1080, and 950 bp) common to strains A through F, mutant strains A and D were the only ones having a band at 600 bp. Mutant strains B, C, and F were similar to each other, differing mainly in the intensity of the bands and in addition to the three bands common to A through F, their profiles also had a band at 1900 bp. Strain E was unique among the mutants because it had only the three bands common to A through F (none at the same location as the three bands in the wild-type strain); all the other mutants had a total of four main bands except B2, which had two main bands (also seen in wild type). SDS-PAGE analysis of the proteins in the mutant and wild-type strains produced similar band patterns for all strains with the notable exception of a band at approximately 34 kD, which appeared strongly only in the pattern for mutant strain A (Fig. 4b), the only strain selected from the plates that underwent 4-hour irradiation. All other strains were taken from plates irradiated for 2 h.

### Growth and doubling times

The optical density values at 660 nm determined for *Y. lipolytica* wild-type and mutant strains cultured in 60 mL of LNG liquid medium in 125-mL flasks incubated at 30 °C for 96 h and the initial doubling times calculated for each strain are given in Table 1. The maximum optical density values were highest for mutant strain B2 (0.869 at 96 h) and next highest for wild-type strain (0.781 at 96 h). The maximum optical density values for the remaining mutant strains, A, B, C, D, E, and F, were lower and similar to each other, 0.622 (72 h), 0.598 (72 h), 0.591 (96 h), 0.594 (96 h), 0.610 (96 h), and 0.613 (96 h), respectively. The doubling times of wild-type strain (1.5 h) and mutant strain F (1.4 h) were shortest of all the strains. The doubling time of mutant strain B2 was 1.6 h. Mutant strains C and E both had a doubling time of 1.8 h, and mutant strains A and B had doubling times of 2.0 h and 2.1 h, respectively. Mutant strain D had the longest doubling time (2.9 h).

**Table 1:** Growth and doubling time of *Yarrowia lipolytica* NRRL YB-567 wild-type (WT) and mutant strains B2, A, B, C, D, E, and F in LNG liquid medium

Time (h)	Optical density at 660 nm (OD_660_)							
	WT	B2	A	B	C	D	E	F
0	0.020	0.024	0.022	0.023	0.020	0.023	0.023	0.020
1	0.027	0.026	0.023	0.023	0.025	0.024	0.030	0.021
1.5	0.027	0.035	0.023	0.025	0.033	0.024	0.038	0.027
2	0.055	0.040	0.040	0.045	0.041	0.030	0.050	0.034
2.5	0.064	0.069	0.052	0.053	0.053	0.042	0.061	0.068
24	0.372	0.367	0.379	0.432	0.386	0.349	0.327	0.353
48	0.621	0.588	0.531	0.506	0.503	0.451	0.430	0.529
72	0.621	0.743	0.622	0.598	0.528	0.596	0.498	0.586
96 (4 days)	0.781	0.869	0.607	0.588	0.591	0.594	0.610	0.613
Doubling Time^a^ (h)	1.49	1.63	2.01	2.08	1.78	2.88	1.76	1.42

^a^Calculated using equation: initial doubling time (hours) = *Δt* [ln 2/ln(*A/A*_*0*_)] where ln = natural log; *A*_0_ = optical density at *t*_0_; *A* = optical density at *t* (2.5 h); and Δ*t* = *t* − *t*_0_

### Ammonia, protein, and 2-phenylethanol levels

**Fig. 5:**
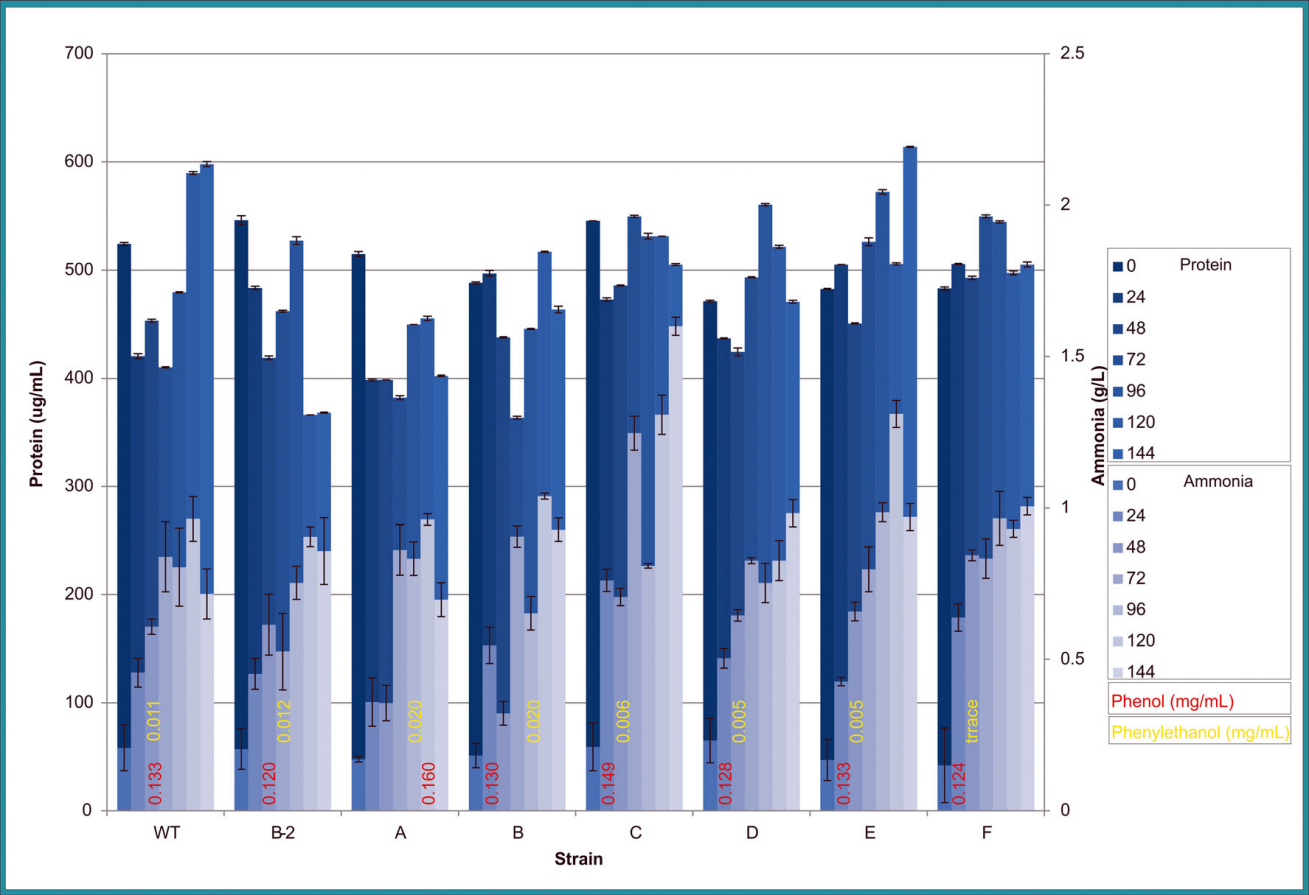
Protein (Bradford Assay) and ammonia (enzyme assay) levels of *Yarrowia lipolytica* NRRL YB-567 wild-type and mutant strains *B2*, *A*, *B*, *C*, *D*, *E*, and *F* grown on LNG medium at 24, 48, 72, 96, 120, and 144 h, with ammonia levels (light blue) superimposed on protein levels (dark blue) and phenol and 2-phenylethanol levels indicated in red and yellow, respectively, at the bottom of the graph

**Fig. 6:**
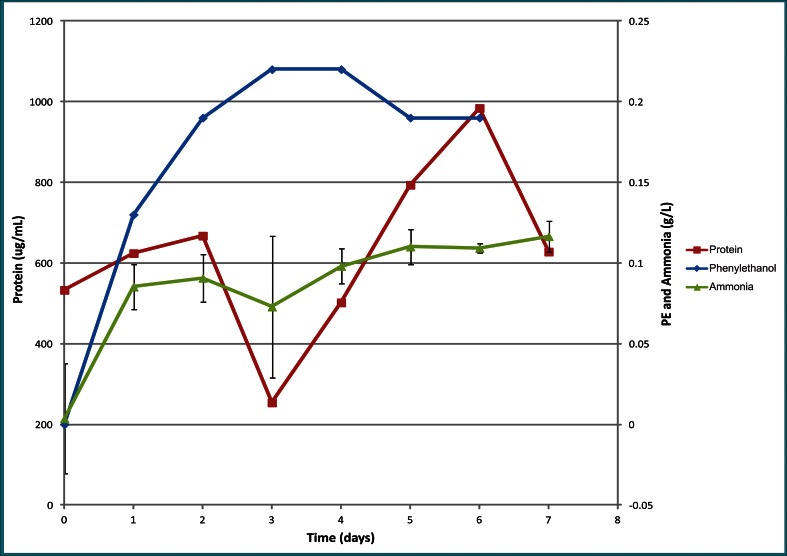
*Yarrowia lipolytica* NRRL YB-567 mutant strain F ammonia (error bars shown for three replicates), protein, and 2-phenylethanol production on *Kluyveromyces marxianus*-treated coffee pulp and mucilage over a 7-day period

Levels of ammonia and soluble protein for wild-type strain and each of the mutant strains when grown on LNG liquid medium for up to 144 h are depicted in Fig. 5. Ammonia levels (light blue) are superimposed on protein levels (dark blue). Ammonia levels over time were highly variable from strain to strain but generally increasing for all strains. The maximum ammonia levels reached for wild-type strain and mutant strains B2, A, B, and E were 0.96, 0.91, 0.96, 1.04, and 1.31 g/L, respectively, at 120 h, and for mutant strains C, D, and F were 1.60, 0.98, and 1.01 g/L, respectively, at 144 h. On *K. marxianus*-treated CWM, the maximum ammonia level of mutant strain F was 0.12 g/L at 7 days (Fig. 6).

Similar to ammonia levels, protein levels over time were highly variable from strain to strain (Fig. 5). Initially, soluble protein concentrations were similar for all strains and ranged from 546 μg/mL (mutant strain B2) to 471 μg/mL (mutant strain D). Maximum soluble protein level was highest for mutant strain E (614 μg/mL at 144 h). For wild-type strain, the maximum protein level was 598 μg/mL at 144 h. Maximum soluble protein levels for mutant strains B2 and A, were 546 and 515 μg/mL, respectively, (0 h), for both mutant strains C and F it was 550 μg/mL (72 h), for mutant strain D it was 560 μg/mL (96 h), and for mutant strain B it was 517 μg/mL (120 h). On *K. marxianus*-treated CWM, the protein level of mutant strain F was 525 μg/mL initially and reached a maximum of 990 μg/mL at 144 h (Fig. 6).

The amount of 2-phenylethanol produced on LNG medium (numbers in yellow on Fig. 5) was highest for mutant strains A and B (0.020 mg/mL). Wild-type strain and mutant strain B produced 0.011 and 0.012 mg/mL 2-phenylethanol, respectively, while mutant strains C, D, E, and F produced less than 0.010 mg/mL. However, on *K. marxianus*-treated CWM, mutant strain F produced a maximum of 0.22 mg/mL 2-phenylethanol at 96 h (Fig. 6).

### Oil production

The wild-type and mutant strains were also evaluated for oil (triglycerides) production using Sudan Black B indicator. The use of Sudan Black B for staining and direct observation under the microscope enables the rapid observation of the qualitative status of lipid production in the cells (Thakur et al. 1988). Sudan Black B is primarily made up of non-polar groups so it partitions into the lipid phase, staining the lipid. As pictured in the photograph in Fig. 1, step 3, mutant strain F produced the darkest black area with this indicator. Densitometry measurements were performed to quantitate the amount of dye bound to each strain. The results, expressed in percent of wild type, for mutant strains A, B2, B, C, D, E, and F were 81.5, 90.6, 89.5, 82.7, 82.2, 81.3, and 148.3 %, respectively, demonstrating that mutant strain F produced 48 % more oil than the wild-type strain on LNG medium.

The fatty acid composition of the oil produced by mutant strain F on *K. marxianus*-treated CWM is presented in Table 2. The composition without the addition of spent coffee grounds (borra) was fairly evenly distributed among palmitic (C16:0), stearic (C18:0), and linoleic (C18:2) acids at 28.5 %, 30.0 %, and 26.5 %, respectively. With the addition of borra, the distribution was shifted toward linoleic with 27.1 % palmitic, 20.0 % stearic, and 36.7 % linoleic. Without borra, 2.38 % of the fatty acids were longer than C18; with borra, 3.45 % were longer than C18, including C20:0, C22:0, and C30:0. More than half the acylglycerols were triacylglycerols.

**Table 2:** Fatty acid composition of lipids produced using *Yarrowia lipolytica* mutant strain F on *Kluyveromyces*-treated coffee waste compared to that for other *Y. lipolytica* strains and to plant oils (references provided in column headings)

Fatty acid	Strain F (without borra^a^) (%)	Strain F (with borra) (%)	Strain W29 in YNBO^b^ medium (%) (Mlíčková et al. 2004)	Strain W29 in wheat straw hydrolysate (%) (Yu et al. 2011)	Strain W29 Po1g in sugarcane bagasse hydrolysate (%) (Tsigie et al. 2011)	Strain MUCL 28849^c^ (%) (Fontanille et al. 2012)	Palm (%) (Bio-diesel 2013)	Soy (%) (Bio-diesel 2013)	Canola (%) (Bio-diesel 2013)
C16:0 = palmitic	28.5	27.1	5.5	5.7 (total of all *C16*)	17.76	14–23	41.1	10.2	4.0
C16:1 = palmitoleic	1.8	8.2	16		14.12	3–8	–	–	–
C18:0 = stearic	30.0	20.0	1.1	0.8	4.39	6–39	5.3	4.2	1.9
C18:1 = oleic	3.7	2.9	66	55.3	55.55	17–37	40.3	21.7	61.9
C18:2 = linoleic	26.5	36.7	8.7	20.9		11–31	10.8	53.1	19.3
C18:3 = linolenic	1.35	0.92	–					7.0	9.3
C20:0 = arachidic or C20:1	1.37 *(C20:0)*	1.5*(C20:0)*	0.7 (*C20:1*)						1.2 (*C:20:1*)
C22:0 = behenic	1.01	0.73							
C30:0 = melissic	0	1.22							
	*94.2*	*99.3*	*98.0*	*82.7*	*91.8*		*97.5*	*96.2*	*97.6*
Monoglycerides	11.0	4.7							
Diglycerides	37.1	33.4							
Triglycerides	52.0	62.0							
	*100.1*	*100.1*							

^a^Borra = spent coffee grounds

^b^YNBO = minimal oleic acid medium containing yeast nitrogen base

^c^On various glucose, glycerol, and volatile fatty acid combination substrates

### Substrate utilization

**Fig. 7:**
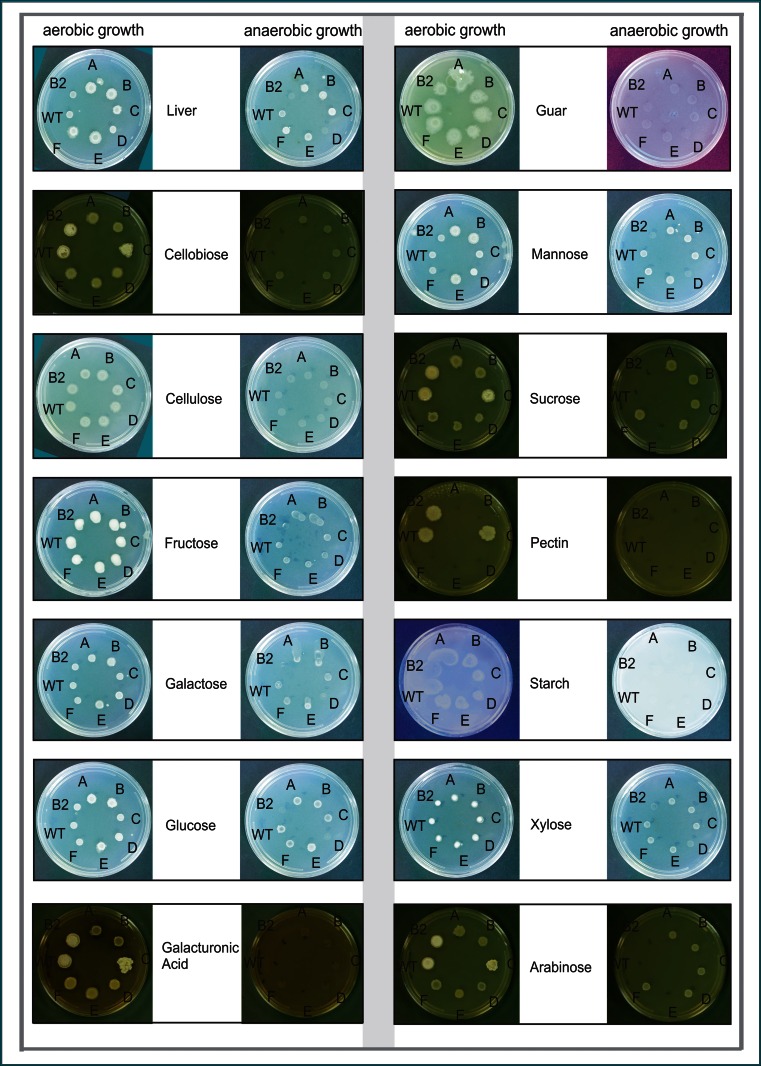
Growth of *Yarrowia lipolytica* NRRL YB-567 wild-type (*WT*) and mutant strains *B2*, *A*, *B*, *C*, *D*, *E*, and *F* on plates containing the carbon sources indicated. Cultures of the strains in liquid LNG medium were adjusted to OD_660_ = 0.5, and 10 μL of each culture were spotted onto each plate and incubated for 2 days at 30 °C

In plate assays, the mutant strains grew on a variety of substrates similar to the wild-type strain as shown in Fig. 7. All strains grew well on LNG medium, with mutant strains A, B, C, E, and F slightly better than B2, D, and wild type. On the polymers guar (galactomannan) or cellulose [glucose units joined β(1→4)], all strains showed moderate growth aerobically but no growth anaerobically. No strains grew on starch [glucose units joined α(1→4)]. On pectin [galacturonic acid units joined α(1→4)], only wild type and mutant strains B2 and C showed weak growth aerobically; no strains grew anaerobically. On the disaccharide cellobiose [glucose joined β(1→4)], all strains showed weak growth aerobically (wild type, B2, and C slightly better) and no growth anaerobically. Growth on sucrose for all strains was faintly detectable aerobically. On mannose, galactose, or glucose, all strains grew aerobically with mutant strains A, B, and E showing very slightly better growth; all strains except B2 and D showed weak growth anaerobically. On galacturonic acid, all strains grew weakly aerobically (wild type, B2, and C slightly better), but did not grow anaerobically. On fructose, all strains grew very well aerobically and showed faint growth anaerobically. On xylose, all strains showed growth aerobically and all strains except B2 showed weak growth anaerobically. On arabinose, all strains showed weak growth aerobically, with wild type, B2, and C slightly better than the other strains; mutant strains A, B, C, D, and F grew very faintly anaerobically, while wild type, B2, and E did not grow anaerobically.

## Discussion

### Production of Yarrowia lipolytica NRRL YB-567 mutant strains

The yeast strain *Y. lipolytica* NRRL YB 567 was selected for the present study because of its ability to utilize protein to produce ammonia (Gardini et al. 2001; Ismail et al. 2000; Kurtzman 2011; Mansour et al. 2008). Using a similar procedure to that described previously for *Kluyveromyces marxianus* NRRL Y-1109 (Hughes et al. 2013), we irradiated *Y. lipolytica* NRRL YB-567 with UV-C to enhance ammonia (fertilizer) and lipid (oil) production on low-cost protein and carbohydrate substrates and screened the resulting mutant strains first for ammonia then for oil production using color intensity of indicator compounds on plate assays. Seven mutant strains, designated A, B, B2, C, D, E, and F, were selected (ammonia assay) and further evaluated for growth rate, ammonia and oil production, and soluble protein content on liver infusion medium (without sugars) and for growth on various substrates. Mutant strain F produced substantially more oil and had a faster doubling time than the other mutant strains. Mutant strain E had a higher maximum soluble protein level than other mutant strains. These two mutant strains, along with mutant strains B and C, produced higher maximum ammonia levels compared to wild type. When grown on plates with substrates of interest, the strains showed similar results aerobically, except that only mutant strains C and B2 retained the ability of wild type to grow on pectin.

### Morphological evaluation

*Y. lipolytica* is a dimorphic fungus, which forms yeast cells, pseudohyphae, and septate hyphae. True mycelia consist of septate hyphae 3 to 5 μm in width and up to several mm in length. Apical cells often exceed 100 μm, whereas segments are 50- to 70-μm long (Barth and Gaillardin 1997; Coelho et al. 2010). *Y. lipolytica* is able to undergo a true yeast–hypha transition depending on the medium. It grows as the yeast form with a polar budding pattern in YNB medium, while hyphal growth can be induced either by replacing glucose with *N*-acetylglucosamine or by adding serum to the culture medium (Nicaud 2012). If the cells are grown on minimal medium supplemented with *N*-acetylglucosamine as the carbon source, a reproducible system for the production of mycelium is obtained and electron microscopy shows mycelium with a smooth surface, a great amount of mitochondria, and a low degree of branching. In complex media, a mixed morphology of yeast and mycelial forms is obtained. Madzak et al. (2005) demonstrated cell morphology (proportion of yeast cells and mycelia) was highly variable depending on the culture medium: at stationary phase, only the yeast cell form was observed in YPD (yeast extract-peptone-D-glucose), or PPB 20 Cit (sucrose-yeast extract-KH_2_PO_4_-NH_4_Cl-MgSO_4_-thiamine/20 mM citrate buffer pH 6), or PPB 20 Ph (PPB/20 mM phosphate buffer, pH 8), or PPB 50 Ph medium, while a mixture of yeast cells and mycelia was obtained in Young medium (glucose-yeast nitrogen base-BSA; Young et al. 1996) or PPB 50 Cit medium, and only the mycelial form was observed in YNB (glucose-yeast nitrogen base without amino acids-(NH_4_)_2_SO_4_-NH_4_Cl-50 mM phosphate buffer pH 6.8) or PPB 200 Cit medium (Madzak et al. 2005). The proportion of the different cell forms probably accounts for the morphological differences observed at colony level. Wild-type strains of *Y. lipolytica* exhibit various colony shapes, ranging from smooth and glistening to heavily convoluted and matte. The colony morphology is determined both by growth conditions (including aeration, carbon and nitrogen sources, and pH) and by the genetic background of the strain (Barth and Gaillardin 1997).

In our study, the morphology of the mutant strains differed from the wild-type strains and from each other. The most notable morphological differences were the much smaller cell size (yeast-like forms about 1–2 μm in length) of mutant strains D, E, and F compared to the other mutant strains, A, B, B2, and C, and to the wild-type strain (yeast forms about 5 μm in length) and the bumpier cell surface of mutant strain F compared to the other strains. The cells of the wild-type strain exhibited both yeast (mostly ellipsoidal) and pseudohyphal forms with the yeast forms similar in size and shape and within the size and shape ranges reported in the literature for *Y. lipolytica* strains (Barth and Gaillardin 1997; Nicaud 2012). The surfaces of the yeast forms were smoother than the pseudohyphal projections, and the cells have budding scars at the ends. Mutant strains A, B, B2, and C consisted of cells of various combinations of shapes and sizes of both yeast and pseudohyphal forms with varying amounts of smooth and bumpy surfaces, with the yeast forms generally in the size range of the wild type. The cells of mutant strains D, E, and F were smaller than those of the other strains. The cells of D and F showed extensive branching and were intertwined forming a unique layer-like assembly. The surfaces of all cells in strain F were very bumpy, possibly caused by numerous oil droplets adhering to the surface as suggested by its high oil content, similar to the scanning electron micrographs of *Y. lipolytica* cells grown in minimal oleic acid medium containing yeast nitrogen base (without amino acids and ammonium sulfate)-NH_4_Cl-yeast extract-oleic acid-50 mM phosphate buffer pH 6.8. (Mlíčková et al. 2004).

### VNTR analysis of genomic DNA

VNTR analysis uses a form of polymorphic DNA found in so-called minisatellite regions which contain variable numbers of tandem repeats (VNTRs). In eukaryotes, these regions consist of short (10 to 100 bp, most commonly 15 bp) sequences of DNA repeated end-to-end at a defined locus in the genome. They can be found on many chromosomes and, while the repeated sequences are usually the same from individual to individual, the number of repeat units at the same locus often varies. Flanking the repeats are segments of non-repetitive sequence, allowing the VNTR blocks to be amplified via PCR, separated by gel electrophoresis, and the resultant DNA fragments compared without the need for special gene probes (Pourcel et al. 2011).

The analysis of variable numbers of tandem repeat loci identified in the genomes of eukaryotic and prokaryotic species during genome sequencing projects has been shown to be a useful technique for the molecular typing of clinical isolates of several bacterial species including *Yersinia pestis*, *Francisella tularensis*, *Bacillus anthracis*, and *Staphylococcus aureus* (Sabat et al. 2003). A review by Lindstedt (2005) further highlights that the variable number of tandem repeats has proven to be a suitable target for assessing genetic polymorphisms within bacterial species. It gives an overview of bacterial agents where VNTR-based typing, or multiple-locus variant-repeat analysis (MLVA) has been developed for typing purposes, together with addressing advantages and drawbacks associated with the use of tandem repeated DNA motifs as targets for bacterial typing and identification (Lindstedt 2005).

Tandemly repeated sequences provide a very valuable source of polymorphism, and MLVA is now used in genotyping several bacterial species. MLVA typing relies upon a basic and widespread methodology, the measurement of the length of DNA fragments. It is not a pattern-producing method, even when run on agarose gels. The genotype is given as a set of numbers corresponding to the number of repeats at each locus (Pourcel et al. 2009).

Agarose gel analysis of the PCR products, amplified from the genomic DNA of *Y. lipolytica* NRRL YB-567 mutant strains using a VNTR primer, produced DNA fragments of different lengths from those of the other mutants and of the wild-type strain, with six of the seven mutants having none of the three bands produced by the wild-type strain and the other mutant strain (B2) having only two of the three wild-type bands. Disappearance of the three bands observed in the wild-type in six of the seven mutant strains indicates that irradiation caused significant changes from wild type in the regions amplified by this VNTR primer in the wild-type, and the appearance of five completely new bands in this region suggests that different mutations were caused in the genomic DNA of wild-type *Y. lipolytica* NRRL YB-567. The differences in the lengths of the DNA fragments for the various *Y. lipolytica* mutants indicate different numbers of repeats resulting from differences in the *Y. lipolytica* genome among the mutant strains and between the mutant strains and the wild-type strain.

### Evaluation of doubling times

Madzak et al. (2005) determined the doubling times of three strains of *Y. lipolytica* (two laccase-producing strains and a non-producing control strain Po1t) in four different liquid media incubated at 28 °C: (1) YPD (yeast extract-peptone-D-glucose), (2) young medium (glucose-yeast nitrogen base-BSA), (3) YNB [glucose-yeast nitrogen base without amino acids and (NH_4_)_2_SO_4_-NH_4_ Cl-50 mM phosphate buffer, pH 6.8], and (4) PPB (sucrose-yeast extract-KH_2_PO_4_-NH_4_Cl-MgSO_4_-thiamine), under different buffering conditions, either with 20, 50, or 200 mM citrate buffer, pH 6 (respectively, PPB 20 Cit, PPB 50 Cit, and PPB 200 Cit), or with 20 or 50 mM phosphate buffer, pH 8 (respectively, PPB 20 Ph and PPB 50 Ph). Aliquots (1 ml) were collected daily, and mean doubling time was determined between days 0 and 1. The results in each medium were similar for all three strains. The doubling times were as follows: 1.7 h in YPD, 2.5 h in YNB, 3.7 h in Young medium, 3.5 h in PPB 20 Cit, 2.4 h in PPB 50 Cit, 2.1 h in PPB 200 Cit, 2.2 h in PPB 20 Ph, and 2.2 h in PPB 50 Ph. Most doubling times ranged from 1.7 to 2.5 h; however, for basic PPB medium, doubling time was much longer (3.5 h) in the lowest concentration of citrate buffer, and doubling time was longest (3.7 h) in the glucose-yeast nitrogen base-BSA medium (Madzak et al. 2005). Titorenko and Rachubinski (1998) determined doubling times for *Y. lipolytica* strain DX547-1A on YPD medium to be 2.4 h at 22 °C and 2.2 h at 32 °C (Titorenko and Rachubinski 1998). *Y. lipolytica* can form either yeast cells or hyphae and pseudohyphae, depending on growth conditions (aeration, carbon and nitrogen sources, pH, etc.). Cell morphology (proportion of yeast cells and mycelia) is highly variable depending on the culture medium. Madzak et al. (2005) determined that in YPD, cells were yeast shaped; in YNB, cells formed mycelia; and in Young’s medium, both morphological forms were present; however, they made no adjustments to doubling time for different morphologies. No correlation between morphology and doubling time was seen in their study.

In our investigation, the doubling times varied with the strains, but were similar to those reported in the literature, although the medium used was different. The doubling times of wild-type strain (1.5 h) and mutant strain F (1.4 h) were shortest of all the strains studied. The doubling time of mutant strain B2 was 1.6 h. Mutant strains C and E both had a doubling time of 1.8 h, and mutant strains A and B had doubling times of 2.0 and 2.1 h, respectively. Mutant strain D had the longest doubling time (2.9 h). No correlation between morphology and doubling time was observed.

### Comparison of ammonia production, protein levels, and 2-PE production

*Y. lipolytica* secretes acidic and alkaline proteases depending on pH of the growth medium. Production is also controlled by carbon, nitrogen, and sulfur starvation. Both proteases are induced at the end of the exponential phase on complex protein-containing media, but the type synthesized depends strictly on pH (Harzevili 2014). *Y. lipolytica* is an ubiquitous yeast that develops on the cheese surface, and its enzymatic activities make it useful in the preparation of cheese flavor compounds. Cheese curd is a complex matrix containing carbon and/or nitrogen sources that are important in ripening, including lactate from the conversion of lactose by lactic acid bacteria and amino acids resulting from casein proteolysis. Mansour et al. (2008) studied the growth of a strain of *Y. lipolytica* isolated from cheese in liquid medium containing lactate in the presence of high or low concentrations of amino acids. Their data suggested that amino acids were used by *Y. lipolytica* as a main energy source, while the lactate is consumed following amino acid depletion. Amino acid degradation was accompanied by ammonia production corresponding to a dramatic increase in the pH (Mansour et al. 2008).

Our objective was to identify a strain capable of producing valuable products from coffee and fruit processing wastes and also from fermentation residues, mainly protein, in a biofuel refinery. *Y. lipolytica* was shown to be capable of using protein substrates to produce ammonia, which is a valuable fertilizer. We irradiated the wild-type strain in an effort to cause mutations that would enhance ammonia production. In our study, ammonia levels for each of the mutant strains when grown on LNG liquid medium for up to 144 h were highly variable from strain to strain. The maximum ammonia levels for four of the mutant strains, B (1.04 g/L), C (1.60 g/L), E (1.31 g/L), and F (1.01 g/L) were higher than for the wild-type strain (0.96 g/L).

Similar to the ammonia levels, the protein levels for each of the mutant strains when grown on LNG liquid medium for up to 144 h were highly variable from strain to strain. Initially, soluble protein concentrations were similar for all strains and ranged from 546 μg/mL (mutant strain B2) to 471 μg/mL (mutant strain D). The maximum soluble protein level was highest for mutant strain E (614 μg/mL) at 144 h, only slightly higher than for the wild type strain (598 μg/mL) at the same time point. No correlation between ammonia production and soluble protein concentration was distinguishable.

2-Phenylethanol is an important fragrance, flavor, and commodity chemical widely used in cosmetics and food products. The synthetic and natural versions differ substantially in value, with synthetic 2-phenylethanol priced around $3.5/kg in the world market (about 7000 tons in 2007) while the material produced by natural routes, from rose petals or microbiological conversion, commands prices up to $1000/kg, with a global estimated market in 2007 of 0.5 to 1 ton per year. The most efficient 2-phenylethanol synthesis process in yeasts is the multi-step bioconversion of L-phenylalanine known as the Ehrlich pathway (Celińska et al. 2013). Celińska et al. (2013) demonstrated that *Y. lipolytica* was capable of producing relatively high titers of 2-phenylethanol. These researchers cultured six different *Y. lipolytica* strains in YPD medium and determined that varying amounts of 2-phenylethanol were produced, with five of the strains producing 0 to 0.04 g/L and the sixth producing 0.24 g/L. If they supplemented the medium with L-phenylalanine, *Y. lipolytica* NCYC3825 produced a final titer of 1.98 g/L at 95 h after all L-phenylalanine was utilized (Celińska et al. 2013). In our results, the amount of 2-phenylethanol produced on LNG medium was highest for mutant strains A and B (0.020 mg/mL). Wild-type and the other five mutant strains produced 0.012 to 0.005 mg/mL, similar to the results reported by Celińska et al. (2013) on non-supplemented YPD medium. In addition, our investigation demonstrated that on *K. marxianus*-treated CWM, mutant strain F produced a maximum of 0.22 g/L 2-phenylethanol at 96 h, suggesting that the use of *Y. lipolytica* strain F on coffee waste as part of an integrated waste removal platform might also be a potential source of this valuable co-product.

### Oil production evaluation

The complexity and multiplicity of genes present in the genome of *Y. lipolytica* enable it to use a wide variety of substrates to accumulate high levels of lipids (mostly triacylglycerols) (Beopoulos et al. 2009). The accumulation of lipids has potential application for production of specific valuable fatty acids (omega-3 oils) (Xue et al. 2013) or for production of non-specific oils for renewable diesel (Xu et al. 2013). Although lipid content and fatty acid profiles differ between species and depend on substrate, on average, the best-known oleaginous yeasts accumulate lipids to a level corresponding to 40 % of dry cell weight (Fontanille et al. 2012), and under some conditions, they may accumulate levels up to 70 % (*w*/*w*) (Beopoulos et al. 2009). For *Y. lipolytica*, lipid levels of 43 % on crude glycerol using *Y. lipolytica* LGAM S(7)1 (Papanikolaou and Aggelis 2002), and of 58.5 % (*w*/*w*) on sugarcane bagasse hydrolysate using *Y. lipolytica* Po1g, a genetic modification of wild-type strain W29 (ATCC 20460) (Tsigie et al. 2011), have been reported. On lignocellulosic biomass, *Y. lipolytica* lipids have a very high percentage of unsaturated fatty acids, for example, 55.3 % oleic (C18:1) and 20.9 % linoleic (C18:2) on wheat straw hydrolysate using *Y. lipolytica* W29 (ATCC 20460) (Yu et al. 2011).

In experimental investigations on the improvement of yields and productivities of lipids from oleaginous microorganisms in fermenters, a bottleneck is the speedy determination of the extent of accumulation of the intracellular lipids. The use of Sudan Black B for staining and direct observation enables the rapid determination of the status of lipid production in the cells (Thakur et al. 1988). Densitometry measurements performed in our investigation using Sudan Black B to determine the lipid content in each mutant strain relative to the wild-type strain demonstrated that mutant strain F accumulated 48 % more oil than the wild-type strain on LNG medium, possibly indicated by the large number of oil-droplet-like bumps on the surface of the cells observed in the scanning electron micrographs. We also determined the fatty acid profile for the acylglycerols obtained from the growth of *Y. lipolytica* mutant strain F on *K. marxianus*-treated coffee waste (Table 2). Our results showed that 85 % of the composition without the addition of spent coffee grounds (borra) was fairly evenly distributed among palmitic (C16:0), stearic (C18:0), and linoleic (C18:2) acids at 28.5, 30.0, and 26.5 %, respectively. With the addition of borra, the distribution was shifted from steric toward linoleic with 27.1 % palmitic, 20.0 % stearic, and 36.7 % linoleic acids. Both profiles contained large amounts of fatty acids with favorable properties for biodiesel (Knothe 2005). Without borra, 2.38 % of the fatty acids were longer than C18; with borra, 3.45 % were longer than C18, including C20:0, C22:0, and C30:0. More than half the acylglycerols were triacylglycerols. This distribution of fatty acids falls in the range of the results for the lipid compositions determined by Fontanille and co-workers (2012) using *Y. lipolytica* MUCL 28849 grown on various glucose and glycerol mixtures with volatile fatty acids (Table 2). The composition was also similar to that of vegetable oils used for industrial production of biodiesel (Biodiesel 2013; Knothe 2005).

### Substrate utilization by wild-type and mutant strains

Currently, utilization of cellulosic biomass including agricultural and food wastes has drawn increasing attention as a means of minimizing the cost of bio-oil production (Tsigie et al. 2011; Xu et al. 2013). *Y. lipolytica* already uses a range of substrates (Goncalves et al. 2014; Kurtzman 2011; Mansour et al. 2008) that makes it a potential organism for converting coffee and fruit processing wastes and spent fermentation residue to biofuels and valuable co-products in a sustainable biorefinery system. Our focus in this study was the development of a mutant *Y. lipolytica* microbial strain with enhanced ability to bioprocess constituents of plant and food biomass, including agricultural waste from cocoa, coffee, manure, energy trees, energy grasses, and food waste to produce a mixture of yeast biomass, bio-oil, and other high-value bioproducts. We evaluated the ability of the seven mutant strains that we generated to grow on some of the compounds that would potentially be constituents of these sources, including arabinose, xylose, fructose, galacturonic acid, glucose, galactose, mannose, sucrose, cellobiose, starch, pectin, cellulose, and guar. The plate test was used to provide a rapid indication of the relative ability of the mutant strains to use these substrates compared to each other and to wild type as well as to see if any mutants displayed good growth on new substrates that were of interest. The results of the evaluation of the growth of these strains on plates containing these compounds demonstrated that, except for the utilization of pectin aerobically, the mutant strains retained the ability to grow as well or slightly better than wild type on these substrates. Only mutant strains C and B2 retained the ability of the wild-type strain to grow on pectin aerobically. Also, mutant strains B2 and D did not grow anaerobically on glucose, galactose, mannose, or xylose, while the other strains all showed weak growth.

A renewable gas/diesel biorefinery process is being developed for which microbial strains will be optimized for industrial conditions and for ability to express genes for advanced biofuel and chemical production (Hughes et al. 2014). In this biorefinery concept, a series of fermentations including mutant *Y. lipolytica* microbial strains and other microbial strains are being designed to bioprocess all carbohydrates and proteins of plant biomass, including agricultural waste from cocoa, coffee, manure, energy trees, energy grasses, and food into a high-solids liquid stream and produce a mixture of yeast cells, oil, biofuels, and other high-value products. The *Y. lipolytica* mutant strains produced in this study warrant further investigation based on successful growth and production of valuable bioproducts on protein, sugars, and coffee waste substrates. The ammonia and oil produced by the novel strains have potential industrial applications such as bio-based fertilizer production to offset the traditional nonrenewable Haber-Bosch process and as renewable drop-in replacements for gasoline via catalytic cracking, diesel via hydrotreatment, or third generation biodiesel fuel via transesterification.
